# Understanding and Improving Older People’s Well-Being through Social Prescribing Involving the Cultural Sector: Interviews from a Realist Evaluation

**DOI:** 10.1177/07334648231154043

**Published:** 2023-02-01

**Authors:** Jordan Gorenberg, Stephanie Tierney, Geoff Wong, Amadea Turk, Sebastien Libert, Caroline Potter, Kathryn Eccles, Shona Forster, Kerryn Husk, Helen J. Chatterjee, Emma Webster, Beth McDougall, Harriet Warburton, Lucy Shaw, Kamal R. Mahtani

**Affiliations:** 1 Nuffield Department of Primary Care Health Sciences, 6396 University of Oxford, Oxford, UK; 2 Nuffield Department of Population Health, 6396 University of Oxford, Oxford, UK; 3 Oxford Internet Institute, 6396 University of Oxford, Oxford, UK; 4 Department of Psychiatry, 6396 University of Oxford, Oxford, UK; 5 Peninsula Medical School, 6633 University of Plymouth, Plymouth, UK; 6 4919 Division of Biosciences University College London, London, UK; 7 Gardens, Libraries and Museums, 6396 University of Oxford, Oxford, UK

**Keywords:** social prescribing, older people, cultural spaces, realist evaluation, semi-structured interviews, link workers

## Abstract

Social prescribing is a non-clinical approach to addressing social, environmental, and economic factors affecting how people feel physical and/or emotionally. It involves connecting people to “community assets” (e.g., local groups, organizations, and charities) that can contribute to positive well-being. We sought to explain in what ways, for whom, and why the cultural sector can support social prescribing with older people. We conducted semi-structured interviews with 28 older people (aged 60+) and 25 cultural sector staff. The following nine concepts, developed from interview data, progressed the understanding of tailoring cultural offers, which came from our previous realist review—*immersion, buddying, café culture, capacity, emotional involvement, perseverance, autonomy, elitism, and virtual cultural offers.* Through tailoring, we propose that older people might experience one or more of the following benefits from engaging with a cultural offer as part of social prescribing—being immersed, psychological holding, connecting, and transforming through self-growth.

**What this paper adds**

• The cultural sector has a role to play in social prescribing for older people, but tailoring is important so that offers are acceptable and accessible.
• Tailoring may be especially required when transferring cultural offers for older people online.
• Older people might want autonomy over how they engage with cultural offers, but at times they might need support or encouragement to try new things.

**Applications of study findings**

• To support the well-being of older people, members of the cultural sector and link workers have to liaise and work together.
• One of the aims of such collaborative working should be to provide tailored cultural offers that are acceptable and accessible to older people as part of social prescribing.
• Tailoring calls for adaptation and flexibility (and adequate resources) to meet the diverse needs of older people.

## Introduction

The physical, social, economic, and emotional consequences of COVID-19 challenged people’s well-being in multiple ways. Older adults, in particular, were identified as being “at risk” from the virus itself and responses to it (Age UK, 2020). Research has suggested that limited human contact during this time contributed to social isolation among older people (Lee et al., 2022). Hence, understanding ways to sustain their well-being is important to explore. Social prescribing represents one approach to addressing this problem.

### Social Prescribing Link Workers

Social prescribing involves connecting people to “community assets”—groups, organizations, clubs, charities—to address their “non-medical” issues (including, but not limited to, loneliness). It takes place in a number of countries across the world (Morse et al., 2022). There are several reviews on social prescribing (e.g., Bickerdike et al., 2017; Calderon-Larranaga et al., 2022; Chatterjee et al., 2018; Husk et al., 2020; Pescheny et al., 2018; Tierney et al., 2020). They highlight the need for further research to understand who social prescribing works for, how, why, and in what circumstances.

In 2019, the National Health Service in England (NHS England, 2019) emphasized the importance of social prescribing as part of personalized care, and funded social prescribing link workers (LWs) to be attached to primary care. LWs come from a range of backgrounds, including healthcare and the voluntary-community sector; they may receive some training relevant to their role (e.g., in active listening, the wider determinants of health), but do not hold a professional qualification as a LW (Morse et al., 2022).

LWs help people to identify their health and well-being priorities, and to develop an action plan; this can include connections to appropriate community assets to assist with their non-medical issues. LWs often meet someone more than once and can use motivational techniques (Sharples, 2019) to encourage them to try new things. In the UK, social prescribing has tended to be provided to adults, although in recent years there has been a move toward expanding services to also support young people.

### Cultural Providers and Social Prescribing

Our previous research suggested that, to be effective, LWs need good up-to-date knowledge of a range of community assets in their local area (Tierney et al., 2020). This may include drawing on the cultural sector, which can support people through “cultural offers” based on dance, music, theatre, heritage engagement, and art (Aesop, 2020; Nikki Crane Associates, 2020; Singing for Health Network, 2021). Providing such support was disrupted during the pandemic, as buildings were closed. Technological alternatives were developed to enable people to interact in new ways. There were also examples of offline provision to avoid excluding people lacking digital access (e.g., posting art packs) (Culture, Health and Wellbeing Alliance, 2020).

Our research aimed to understand how the cultural sector supports older people’s (aged 60+) well-being as part of social prescribing, particularly in light of challenges encountered due to COVID-19. We wanted to develop recommendations for the cultural sector about being “referral-ready” (O’Neill, 2010) for social prescribing with older people. To ensure we were able to complete the research in a timely way, and using data from our previous research in this area (Turk et al., 2020), we centered data collection on specific cultural provision—public/curated gardens, libraries, and museums. However, feedback on our findings from stakeholders suggests that our ideas relate to cultural settings more broadly.

## Research Design

We set out to address the question: *Cultural institutions as social prescribing venues to improve older people’s well-being in the context of the COVID-19 pandemic: What works, for whom, in what circumstances, and why?* By well-being we were interested in how satisfied someone is with their life and feeling that it is worthwhile, their daily emotional experiences (e.g., anxiety or happiness), their sense of security (e.g., financial and housing), and physical status.

Our research program included the following elements:

• Detailed stakeholder and public involvement (online meetings).
• A rapid realist review of exiting literature to understand how social prescribing in cultural venues can support older people (Tierney, Libert, et al., 2022).
• A questionnaire completed by LWs to understand how they are (or are not) connecting with cultural providers as part of social prescribing (Tierney, Potter, et al., 2022).
• Interviews with older people and cultural sector staff (described in this paper)—data collection was underpinned by the following secondary research questions:
  • What do older people find helps or hinders them from using cultural organizations, especially in the context of COVID-19, for well-being?
  • What are the challenges and potential solutions for cultural sector staff in providing social prescribing opportunities in the context of COVID-19?

### Realist Research

Realist research was used to address our research question (Pawson & Tilley, 1997); a theory-driven approach that supports the identification of causal factors through the iterative development of a program theory (Pawson, 2013). A program theory involves mapping assumptions about how an intervention operates and developing context-mechanism-outcome configurations (CMOCs) to explain why, when, and for whom it may or may not work. Supplementary file 1 provides definitions of key terms associated with realist research.

### Program Theory

Interview data helped us to refine the program theory we developed from our previous realist review (Tierney, Libert, et al., 2022); it centered on the idea of “tailoring.” Tailoring involves delivering support or treatment in line with someone’s needs and preferences to improve outcomes and experiences of care (Kreuter & Skinner, 2000). It recognizes that a “one-size-fits-all” approach is not appropriate as people differ on things like information required and expectations of their involvement in care (Dekkers & Hertroijs, 2018). In our review, tailoring referred to the fashioning of a cultural offer and information about this to meet the needs of an older person, whilst also accommodating environmental and social circumstances (e.g., social distancing due to a pandemic).

## Methods

We used interview data to confirm, refute, or refine (i.e., “test”), where necessary, the program theory we developed from our realist review (Tierney, Libert, et al., 2022). Using interview data in this way is common within realist evaluations (Manzano, 2016; Mukumbang et al., 2020).

### Sample

We recruited older people using contacts (e.g., individuals we knew running cultural activities) and health or community organizations. They sent out an email or flyers about the study to older people. Cultural sector staff members were recruited using contacts in the field and specific professional bodies. They were sent an email about the study. Anyone interested in taking part contacted the research team for an information sheet. Purposive sampling aimed to achieve variation in the sample to gather a range of perspectives (see Table 1). The sampling criteria related to the review we had undertaken in terms of the age of older people approached and the place of work of cultural sector staff**.** The sample size was informed, to some extent, by the time we had available to conduct this project. However, toward the end of data collection we were not learning vastly new things to inform the program theory, so had reached a point of data redundancy.

**Table 1.** Criteria that Informed Sampling.

Older People	Cultural Sector Staff
• Aged 60 years and older	• Employed in a garden, library, or museum
• Able to give informed consent	• Varied in terms of the venue where they worked, job title and level of seniority, and how long they had worked in the cultural sector
• Those who had and had not visited/used a garden, library, or museum in the past 18 months	
• Able to converse in English	

### Data Collection

Interviews were conducted between March–July 2021 by telephone or Microsoft Teams. Two male social scientists, experienced in qualitative research, carried out the interviews (JG and SL); the former has an interest in the arts and well-being, the latter in the well-being of older people. Each interview lasted between 30 and 60 minutes. Interviews were semi-structured, enabling researchers to follow-up areas of interest not listed on the topic guide (which was informed by the program theory produced from our review). The questions asked sought to further our understanding of how, when, and why the cultural sector can support older people as part of social prescribing (see supplementary file 2 for example questions). Proposed questions were shared with the study’s Patient and Public Involvement group in advance of conducting interviews, who helped to ensure the wording was understandable. Interviews were audio-recorded (with participants’ consent) and transcribed verbatim for analysis.

### Data Analysis

One researcher (JG) was responsible for data from older people, the other (SL) for data from cultural sector staff. Using a realist logic of analysis, they explored how interview data might expand/refine the program theory from our review. The qualitative computer program NVIVO was used to assist data management; the researchers were able to label sections of data as relating to context and/or mechanism and/or outcome within NVIVO. Some codes they used in NVIVO came from our review program theory (Tierney, Libert, et al., 2022). New concepts were also developed; researchers created code labels to summarize what a piece of data was about if it did fit into the existing program theory. The two researchers had regular meetings (fortnightly) to discuss how they were coding data. They discussed CMOCs with other members of the research team, who have backgrounds in researching social prescribing, the cultural sector, general practice, and digital humanities. They also shared them at online stakeholder meetings attended by older people, LWs, and cultural sector representatives.

### Ethical Considerations

Approval was granted by the University of Oxford’s Central University Research Ethics Committee (ref: R73809/RE001). Verbal consent was obtained from interviewees.

## Results

Interviews were conducted with 28 older people, aged 62–80 years; 18 were female and 10 were male. They came from rural and urban parts of one county in England. Of these participants, 21 identified as White British, 3 as Irish, 1 as Mixed White, 1 as Nigerian British, and 1 as Huayi (this information was missing for one participant). We recruited 25 cultural sector staff, aged 25–65 years; 23 were female and 2 were male. Most worked at a museum/gallery (*n* = 17), although 5 participants were from a library, 2 from a garden setting, and one worked across gardens, libraries, and museums. They held diverse roles, ranging from gift shop worker to program manager. They came from organizations based in different parts of England—the north, the midlands, and the south.

### Concepts from the Interview Data

Interviewees shared a wealth of information. Within this paper, following the guidelines for reporting realist evaluations (Wong et al., 2016), we advance the program theory we initially developed from our realist review. The nine concepts reported below (starting with immersion) are those that were salient in terms of helping us to extend our program theory and relevant to the research questions. Supplementary file 3 highlights CMOCs developed in our realist review and how they were extended by the interview data. When describing the concepts developed from interviews (see below), quotations from older people are identified by the letter P and cultural sector staff by CS, followed by their study identification number.

#### Immersion

Evident during interviews was the importance of being immersed in one’s surroundings through the beauty of a curated space and sensory engagement. With botanic gardens, older people indicated how being outside triggered feelings of peace and relaxation. They recounted how colors, fragrances, weather, and scenery all contributed to a feeling of tranquility that would temporarily distract them from daily problems:

“I like the quietness, sense of peace and serenity that I get there… In gardens, I’m interested in the plants – I don’t know a lot about plants, but I enjoy seeing what other people have achieved...” **P16**

Older people tended to focus on the ambiance of botanic gardens, but some also mentioned other venues with peaceful atmospheres, like museums. They described a sense of discovery and satisfaction because they were using their minds through engaging with culture. This enabled them to counter negative associations with aging and bolstered their self-esteem:

“I like going to exhibitions for…I suppose intellectual stimulation or just to feel that I haven’t forgotten about my art history roots completely.” **P19**

They suggested that stimulation, whether through the senses or intellect, in a cultural venue, could distract them from their concerns, and that the stimulation afforded by a curated space was qualitatively different to passive forms of entertainment, such as television watching.

#### Buddying

A common suggestion made by older people we interviewed was having a “buddy” scheme. They said this may be especially important when someone first attends a cultural offer, which can be daunting if unfamiliar with a venue or organization. Participants thought that having another person there to accompany them would be a useful way to feel more at ease and accountable to turn up:

“If you get paired up with somebody to do something, maybe a stranger, might become a new friend, then that sense of you might let somebody else down I think would be quite strong…it’s having that second person who’s got some enthusiasm for it and you can go with them.” **P3**

The underlying characteristics a buddy should have, according to participants, were kindness and understanding. Older people suggested a buddy would need to be relatable; someone they could interact with as an equal.

#### Café Culture

Our realist review established that connecting was a benefit of cultural institutions as part of social prescribing. Interviews extended this understanding and emphasized the importance of cafés as spaces for connection. Cafés provide a pleasant ambiance where people can meet friends or rest before, during or after exploring a venue. Even if there alone, some interviewees said that being in a café surrounded by others made them feel part of a wider community. Participants identified cafés as prime routes for socialization and relaxation:

“…one of the things that I look for in a museum is a café because after wandering around museums, however big they are, big or small, I get quite tired, so I want to sit down and have a cup of coffee and that’s good socially…” **P2**

#### Capacity

It was noted that older people who engaged in social prescribing activities might come to rely on cultural providers to meet their psychosocial needs, or an organization might lack the level of support required for certain individuals:

“…what’s also a concern is saying to someone that you can do this and then not being able to offer the right amount of support, I think is always a real worry. I wouldn’t want us to fail somebody in that way.” **CS19**

Cultural sector staff members wanted to be part of social prescribing but were unsure where their involvement would end and the implications for users and for staff well-being. They stated it was important for social prescribing schemes to have clear exit pathways and boundaries, so older people engaged in a cultural offer did not feel abandoned when it ended.

Some cultural staff members were unsure if they had LWs in their area or how to make contact if they did. For those with direct experience of social prescribing, the importance of working closely with LWs to make sure they received appropriate referrals was noted, although this could take time:

“…link workers are often part of the primary care network…This is quite tricky for museums because often we have small teams…working on a project that is part of our core gallery programme and it’s just hard to get out there if there isn’t a lot of resource to do that.” **CS5**

#### Emotional Involvement

Cultural sector staff may encounter difficult emotions as they support people who are referred as part of social prescribing. Those we interviewed indicated that it could be draining for staff to attend to potentially high needs and expectations:

“This is something I find every single session. No matter how many I do, it’s completely exhausting… it’s a little bit like driving for a really long time because you’re having to keep your attention in so many different places…for such a long period time.” **CS21**

They advised having peer support structures, like debriefing sessions after activities, or access to the external services of a psychosocial/medical professional. They felt this would facilitate the delivery of quality cultural offers by enabling staff to re-energize and recharge emotionally.

#### Perseverance

Continuing with a cultural offer if it does not initially meet immediate expectations was a recurring topic in interviews with older people and cultural sector staff. Participants discussed the importance of a LW communicating clearly to an older person about the cultural offer and its potential benefits, warning them that these benefits may not happen immediately. Interviewees suggested that LWs must demonstrate their credibility by showing they had thought through why a cultural offer might be relevant to an individual’s situation. It was noted that finding an offer that works best for someone may involve experimentation:

“…you’d have to…try to interrogate why it was that it hadn’t been a success and what elements…had been positive and then try and think again…I don’t think you should coerce people into doing it but I suppose if somebody had a very negative attitude towards it, then you might just feel that they should be more open-minded.” **P18**

Perseverance also related to some cultural sector staff’s experience of trying to connect with LWs, to let them know about support they could offer as part of social prescribing:

“…all of the link workers…they’ll have a list of what people can be involved in but I feel like at the minute, we’re still early stages, still shouting about what we doing. We’re still trying to prove…like advertise basically this is what we’re doing. Please use us.” **CS7**

#### Autonomy

Older people we interviewed expressed a strong interest in having control over how they managed their time when engaging with a cultural offer:

“Being able to choose when to go is really important rather than being given a time when you have to clock in…you can build your day around it, or else you can just wander in without having planned anything. Maybe as you get older, sometimes that’s what you need – you need to be able to just do things on a whim.” **P28**

When asked whether they liked going at their own pace or participating in a more structured cultural activity, many older people said they preferred the former because they could choose where to go, what to see, and for how long. Some participants disliked too much structure because it interfered with how immersed they became in an activity. This seemed to be the view for online as well as in-person activities.

#### Elitism

Interviewees noted that museums and other cultural spaces may not always be perceived as welcoming and inclusive; both cultural sector staff and older people said that cultural spaces can be seen as reserved for particular groups of individuals:

“…a preconception [is] that libraries are a kind of…if you're from a traditional working-class background then maybe they're not for you.” **CS22**

“All libraries, museums and galleries are all middle-class pursuits.” **P4**

#### Virtual Cultural Offers

Older people we talked to regarded online offers as qualitatively different to in-person provision; they noted that if someone expected the same experience online they might be disappointed. They acknowledged that virtual offers can expand the options available to LWs and things for older people to try but they had to be engaging and professionally delivered; they needed to be older-people friendly in terms of accessibility, and uncomplicated to use. Having accessible virtual offers extended to speakers and people facilitating events, who interviewees suggested should be familiar with the technology and how to present online:

“…doing talk-overs is not the same as standing in front of an audience. You need to think about how good your energy is – all of those kind of things…I think there could be training courses in how to do presentations via Zoom.” **P23**

While some older participants enjoyed virtual offers, others had no interest, preferring in-person events or activities. Virtual offers were experienced by these individuals as “disembodied” since people attending were not physically together. They described a tangible loss with being online as senses were not engaged in the same way. What some found valuable was having the capacity to socialize on virtual platforms (such as Zoom) using breakout rooms. Yet it was mentioned that interacting online was challenging if not everyone was familiar with a digital platform.

### Revised Program Theory

**Figure 1.**
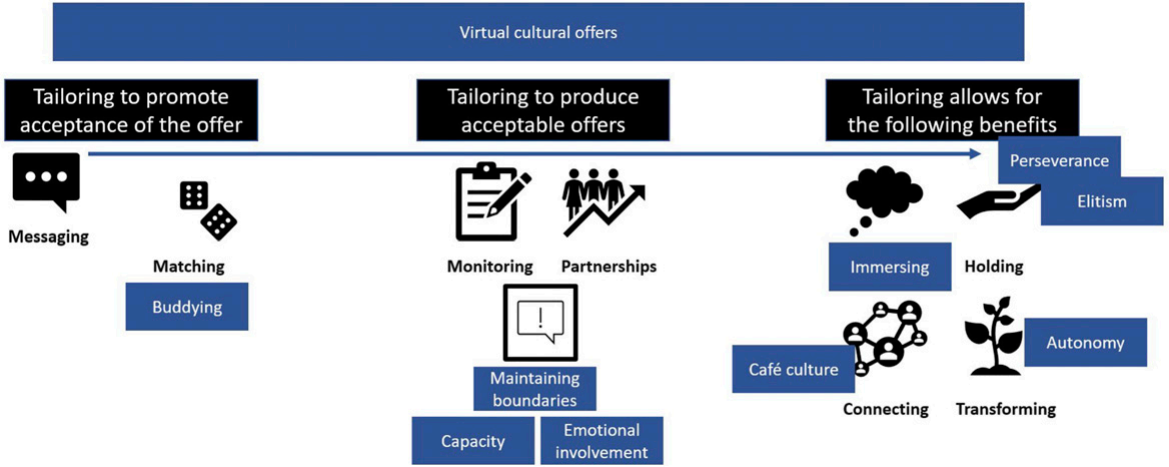
Revised program theory (building on our realist review). It highlights the different elements that need to be considered when tailoring a cultural offer as part of social prescribing to help produce the broad benefits that are represented in the far right of the figure (becoming immersed, feeling psychologically held, making connections, or transforming through self-growth). The blue sections in rectangles have been added or amended based on concepts developed from interview data that are reported in this paper.

The nine concepts presented above helped us to refine the program theory we developed in our realist review, as illustrated in Figure 1. Below, we identify six specific ways that these concepts were incorporated into our revised program theory.

1. The term “distracting” used in our original program theory was replaced with “immersing”; the former term was seen to suggest a more passive encounter with a cultural sector offer. “Immersing,” within the program theory, highlights that providers need to consider the ambiance and atmosphere of a cultural offer, as well as activities or interactions.
2. “Buddying” and “café culture” were concepts developed from interviews with older people. As shown in Figure 1, they should be considered by cultural organizations if planning to provide social prescribing offers. Consideration should also go toward addressing perceptions of “elitism” associated with cultural offers; having a “buddy,” who is regarded as accessible and approachable, may help with this.
3. The idea of “perseverance” was present within interview data. LWs should advise older people about the possible need to persevere when connecting them to a cultural offer; an arrow has been added to the program theory from “messaging” to “perseverance” to illustrate this (see Figure 1).
4. Older people’s wish to be able to shape their experience with the cultural sector appeared to be significant to them getting the most from this encounter and to developing their skills or sense of self. Hence, the notion of “autonomy” has been added to the program theory as important to enabling people to “transform” (e.g., grow in confidence and expand their knowledge) through engaging with a cultural offer.
5. The new concept of “maintaining boundaries” was added to the program theory following the interviews. Cultural sector staff talked about navigating difficulties they faced due to issues of “capacity” (time, funding, and resources) or “emotional investment.”
6. “Virtual cultural offers” is a cross-cutting concept in Figure 1 as it interacts and overlaps with existing components of the program theory. Interview data highlighted that there is potential for virtual offers to help people build their sense of purpose and confidence, as well as developing skills or learning new things, which can be transformative. However, it was suggested that some older people may need support and encouragement to use such offers; this may be provided by LWs or a buddy. Challenges experienced using online offers could disrupt a person’s enjoyment and immersion.

Interview data helped us to further understand the points at which tailoring may be important—from how a cultural offer is presented to an older person by a LW through to the design of offers in the cultural sector that might form part of a social prescription. This is reflected in Table 2, which illustrates broad areas to consider when tailoring a cultural offer as part of social prescribing (see supplementary file 4 for supporting interview data).

**Table 2.** Elements of Tailoring Identified from the Research (see Supplementary File 4 for Supporting Data from Interviews).

**Messaging**	This relates to how the idea of a cultural offer is presented to an older person and is linked to their non-medical needs. This may need to be tailored in a way that encourages someone to be receptive to what is being suggested. The cultural sector needs to provide clear information to LWs about what it can provide so they can talk to patients about such provision in a convincing and knowledgeable manner.
**Matching**	Tailoring may call for LWs to take time to understand what an individual might be open to trying in order to make appropriate connections to cultural offers. They have to know what a cultural offer involves and how it might benefit someone. If a LW or older person perceives cultural offers as “elitist,” this may be a barrier to successful matching. Tailoring in this respect may involve addressing concerns an older person has with trying a cultural offer (this may be done by the LW or by cultural providers). It also relies on having a range of cultural offers available within a local area. Tailoring may entail matching an older person with a “buddy” who can try cultural offers with them (in-person or online).
**Monitoring**	Collecting regular feedback is important to understand if and in what ways cultural offers are accessible and appropriate, and to identify areas for improvement in what is offered and how an offer is presented to an older person. Cultural sector staff should gather such feedback from older people and LWs, and LWs should have catch-ups with older people to see if a cultural offer is working for them. Monitoring in this way enables cultural offers to be tailored to the preferences and needs of specific populations. It could provide information to LWs that would enable them to propose the idea of a cultural offer to older people in a knowledgeable and convincing manner, if they have positive data or testimonials to draw upon. Monitoring data could also be collected so that LWs and cultural providers become aware if certain individuals do not use cultural offers and, if so, to examine why.
**Partnerships**	Positive interactions and relationships between different parties (older people, LWs, and cultural sector staff) are key to tailoring. This can help with the coproduction of accessible and acceptable cultural offers, presented in a way that makes them appealing and able to support older people’s non-medical needs. The more connections a LW has with the cultural sector, the more options they can propose to older people they see; this will mean they can tailor a social prescription to someone’s preferences/needs. Partnerships within a cultural organization are also required so that staff involved in delivering cultural offers feel well supported by their employers. This relates to the idea of having boundaries.
**Maintaining boundaries**	Cultural sector staff described the need for clear exit plans or routes to other support following a social prescribing cultural offer. This has implications when it comes to tailoring. Cultural staff worried that they might lack the skills or infrastructure to assist people with significant psychosocial difficulties. Having space and opportunities to discuss emotional issues arising from social prescribing work was seen as essential for staff we interviewed. Such support may need to be tailored to individual staff needs or the specific offer they are providing as part of social prescribing.

Successful tailoring can help with producing the benefits outlined in Table 3, which we identified from our data as emerging when older people engage with a cultural offer; they may experience one or more benefits, or none if the offer is not tailored to their needs. Some benefits may be quick to arise but short lived (“immersing”), others may be slower to transpire but more profound (“transforming”) (see supplementary file 5 for supporting interview data).

**Table 3.** Potential Benefits that Might Transpire for an Older Person Engaging with a Cultural Offer (see Supplementary File 5 for Supporting Data from Interviews).

**Immersing**	A cultural offer is something that allows people to become absorbed so they are taken away from their problems; it can provide them with immediate relief. It gives them the chance to be present and engaged, to focus on something in the moment. This is possible online or in-person, with learning, knowledge, or an activity stimulating people’s senses. Older people mentioned that online provision needs to be professionally delivered, with funding, skills, staff time, and training provided to make any digital engagement seem like a worthwhile and absorbing experience.
**Holding**	Cultural settings can be places of refuge and comfort where individuals feel psychologically held—accepted and valued. People may need to be encouraged to persevere with a cultural offer by a LW (or a “buddy”), especially if unfamiliar with such spaces or with using online provision. Trying something more than once may be important as benefits may not be immediate; people may be more likely to do this if the cultural offer feels safe and welcoming.
**Connecting**	Gains from engaging in cultural activities can come through interacting with staff and with other people who are present (in-person or online). Replicating a space online where people feel able to connect requires specific consideration, especially for provision aimed at individuals who are unfamiliar with digital interactions. That said, our research showed how for some older people, the pandemic prompted them to become more familiar with online platforms (e.g., Zoom).
**Transforming**	Data suggested there is capacity for self-growth through engaging with cultural offers, which can result in a cognitive shift in how people perceive themselves and their abilities. Such engagement can empower people to feel they have agency and give them confidence to make changes in their life. Having some control over how cultural offers are interacted with (e.g., choices in what activities are undertaken, at what time, in what order, and what items are viewed) might assist with such personal transformation. Volunteering within a cultural setting could also help here, as people get validation and feel valued from undertaking such unpaid work. However, our data suggested that volunteering in cultural settings was more difficult during the pandemic, when venues were shut for sustained periods of time.

## Discussion

Data presented in this paper builds on a previous review we conducted (Tierney, Libert, et al., 2022); it enabled us to test and expand our program theory. We talked to a range of people and gathered information from users of and staff from the cultural sector. They provided valuable insights into how the cultural sector could support older people’s well-being and address their non-medical needs as part of social prescribing. During interviews, older people talked about feelings and the sensory experiences that can come from engaging with cultural offers. Cultural sector staff described more logistical challenges that might occur when involved in social prescribing. Interview data allowed for a more nuanced appreciation of the research topic, which meant additions/refinements were made to the program theory (presented in blue in Figure 1). Rigor and limitations associated with the research are outlined in Table 4.

**Table 4.** Rigor and Limitations Associated with the Research.

Rigor	Limitations
In line with guidance from Lincoln and Guba (1985), we have addressed credibility by having a range of researchers and stakeholders involved in considering the data; dependability by using a clear approach to analysis and coding of data (within NVIVO); transferability by describing individuals involved in conducting interviews, and developing a program theory that draws on not just the interview data but also the review we conducted; confirmability by including quotations from interviewees.	The older people we talked to were mainly White British, from a single county in England, and had not been involved in social prescribing themselves (although they did report using cultural venues for health and well-being purposes). Interviews were conducted remotely because data were collected during the COVID-19 pandemic. This may have meant that some people did not take part. It may also have affected the rapport that researchers were able to build with participants or for them to use and respond to body language. However, participants shared a range of ideas and experiences even though not talking to a researcher face-to-face.

### Connecting Findings to the Existing Literature

Immersion was introduced as a concept in the revised program theory to indicate an engaged experience with a cultural offer, often through people’s senses. This links to previous research on nature-based social prescribing; it has been described as offering the opportunity to smell, touch, taste, and observe in a quiet environment, away from daily stresses, which encourages people to be present in the moment (Garside et al., 2020). Likewise, object handling with museum artefacts can stimulate older people’s senses and produce positive benefits by deflecting their thoughts from health concerns (Thomson & Chatterjee, 2016).

The notion of buddying, which we added to the program theory, is not novel in social prescribing (Hanooman, 2021; Husk et al., 2020). It calls for LWs and/or cultural providers to find buddies who are a good match for individuals referred to an activity. Buddies might benefit from training, to optimize the assistance they provide to those engaging in social prescribing (Pickett, 2021; The Arts Development Company, 2022).

Our data highlighted that older people should be encouraged to persevere with a cultural offer if it does not help immediately, something other researchers have noted (Lovell et al., 2021). Providing a space (in-person or online) where older people feel safe, valued, and welcomed is important, so they want to keep returning (Weiner, 2014). Having some autonomy in how they engage with a cultural offer may encourage such perseverance. The ability to shape their experience within the cultural sector appeared to be significant to interviewees getting the most from this encounter, which has been raised in other research (Watson et al., 2021).

The new concept of maintaining boundaries was added to the program theory because how far tailoring is possible is not unlimited (financially or emotionally). Boundary setting may be something that cultural sector staff members have to establish for themselves to cope with pressures they experience from social prescribing involvement. A report from the Arts Council England (2022) on creativity, culture, and well-being emphasized that practitioners in the cultural sector should be regarded as complementary to not a replacement for health interventions. Receiving permission from their organization to manage competing expectations on them from different actors (e.g., older people, LWs, and policy makers) appears to be important for these staff members.

Digital provision became a key means of enabling people to continue engaging with cultural offers during the COVID-19 pandemic (Cristea & Mutebi, 2022). When undertaking our realist review, we did not find much existing literature specifically on this topic of virtual cultural offers for older people as part of social prescribing. Hence, insights we provide in this paper make a unique contribution to knowledge. However, there are papers on virtual delivery by museums (Beauchet et al., 2022; Koebner et al., 2022), libraries (Ayeni et al., 2022), and gardens (Bettelli et al., 2019); they highlight that such provision can potentially increase accessibility and scalability, alongside improving well-being outcomes. A review of the literature on remotely delivered interventions for older people highlighted that change (or transformation) comes from active rather than passive consumption, with approaches that are adapted (tailored) to meet an individual’s needs and goals (Gorenko et al., 2021). It also emphasized the importance of co-production—involving older people in developing such interventions. Our research supports these ideas.

Designing virtual cultural offers requires good IT support so they are experienced by older people as professionally delivered. Older people may not regard digital provision as relevant or associate it with their interests (Centre for Ageing Better, 2018). Assisting them to see the personal applicability of digital resources may be something that LWs have to undertake when introducing the idea of a virtual cultural offer as part of a social prescription.

### Practical Implications

We have used our refined program theory to draw out a range of practical implications arising from our project (see Table 5). Social prescribing is being implemented in a number of countries (Morse et al., 2022). Therefore, these practical implications will have broad applicability for LWs and cultural providers.

**Table 5.** Practical Implications to Come from the Research.

• Creating a buddy system so an older person has someone to go to a cultural offer with initially; this may be for in-person or online provision. Researchers could explore whether this affects uptake and/or experience of the cultural offer.
• Providing taster sessions of different cultural offers, so older people can try and work out what they might benefit from and enjoy doing/attending.
• Delivering a range of activities within a cultural offer that address different senses and enable people to do or produce or learn about different things.
• Allowing some degree of autonomy in a cultural offer so that people can engage with it in a way that suits their preferences and gives them a feeling of agency.
• Identifying people in an organization who want to be involved in providing cultural offers and investing in training and support for them. Researchers could investigate whether this helps with the emotional involvement and capacity issues reported by staff we interviewed.
• Planning how to make older people feel psychologically held from the moment they engage with a cultural offer. This might include having someone on hand to allay any fears, to reassure and to answer an older person’s questions.
• Having a consistent and simple approach to monitoring, allowing older people to feedback on their experiences of cultural offers (to LWs and the cultural sector).

## Conclusion

Our research highlights that tailoring cultural offers to meet the needs of older people as part of social prescribing is important for them to experience benefits. Interview data extended our previous understanding of tailoring. Interviewees suggested that the ability to tailor is driven by a range of factors including resources and relationships. The cultural sector has a role to play in social prescribing for older people, but this needs careful planning and execution, and good interaction between LWs and cultural sector staff. This seems to be especially the case when transferring offers online, which need to be reshaped and created specifically for a virtual platform.

## Supplemental Material

Supplemental Material—Understanding and Improving Older People’s Well-Being through Social Prescribing Involving the Cultural Sector: Interviews from a Realist Evaluation Click here for additional data file. Supplemental Material for Understanding and Improving Older People’s Well-Being through Social Prescribing Involving the Cultural Sector: Interviews from a Realist Evaluation by Jordan Gorenberg, Stephanie Tierney, Geoff Wong, Amadea Turk, Sebastien Libert, Caroline Potter, Kathryn Eccles, Shona Forster, Kerryn Husk, Helen J. Chatterjee, Emma Webster, Beth McDougall, Harriet Warburton, Lucy Shaw, and Kamal R. Mahtani in Journal of Applied Gerontology.

Supplemental Material—Understanding and Improving Older People’s Well-Being through Social Prescribing Involving the Cultural Sector: Interviews from a Realist Evaluation Click here for additional data file. Supplemental Material for Understanding and Improving Older People’s Well-Being through Social Prescribing Involving the Cultural Sector: Interviews from a Realist Evaluation by Jordan Gorenberg, Stephanie Tierney, Geoff Wong, Amadea Turk, Sebastien Libert, Caroline Potter, Kathryn Eccles, Shona Forster, Kerryn Husk, Helen J. Chatterjee, Emma Webster, Beth McDougall, Harriet Warburton, Lucy Shaw, and Kamal R. Mahtani in Journal of Applied Gerontology.

Supplemental Material—Understanding and Improving Older People’s Well-Being through Social Prescribing Involving the Cultural Sector: Interviews from a Realist Evaluation Click here for additional data file. Supplemental Material for Understanding and Improving Older People’s Well-Being through Social Prescribing Involving the Cultural Sector: Interviews from a Realist Evaluation by Jordan Gorenberg, Stephanie Tierney, Geoff Wong, Amadea Turk, Sebastien Libert, Caroline Potter, Kathryn Eccles, Shona Forster, Kerryn Husk, Helen J. Chatterjee, Emma Webster, Beth McDougall, Harriet Warburton, Lucy Shaw, and Kamal R. Mahtani in Journal of Applied Gerontology.

Supplemental Material—Understanding and Improving Older People’s Well-Being through Social Prescribing Involving the Cultural Sector: Interviews from a Realist Evaluation Click here for additional data file. Supplemental Material for Understanding and Improving Older People’s Well-Being through Social Prescribing Involving the Cultural Sector: Interviews from a Realist Evaluation by Jordan Gorenberg, Stephanie Tierney, Geoff Wong, Amadea Turk, Sebastien Libert, Caroline Potter, Kathryn Eccles, Shona Forster, Kerryn Husk, Helen J. Chatterjee, Emma Webster, Beth McDougall, Harriet Warburton, Lucy Shaw, and Kamal R. Mahtani in Journal of Applied Gerontology.

Supplemental Material—Understanding and Improving Older People’s Well-Being through Social Prescribing Involving the Cultural Sector: Interviews from a Realist Evaluation Click here for additional data file. Supplemental Material for Understanding and Improving Older People’s Well-Being through Social Prescribing Involving the Cultural Sector: Interviews from a Realist Evaluation by Jordan Gorenberg, Stephanie Tierney, Geoff Wong, Amadea Turk, Sebastien Libert, Caroline Potter, Kathryn Eccles, Shona Forster, Kerryn Husk, Helen J. Chatterjee, Emma Webster, Beth McDougall, Harriet Warburton, Lucy Shaw, and Kamal R. Mahtani in Journal of Applied Gerontology.

Supplemental Material—Understanding and Improving Older People’s Well-Being through Social Prescribing Involving the Cultural Sector: Interviews from a Realist Evaluation Click here for additional data file. Supplemental Material for Understanding and Improving Older People’s Well-Being through Social Prescribing Involving the Cultural Sector: Interviews from a Realist Evaluation by Jordan Gorenberg, Stephanie Tierney, Geoff Wong, Amadea Turk, Sebastien Libert, Caroline Potter, Kathryn Eccles, Shona Forster, Kerryn Husk, Helen J. Chatterjee, Emma Webster, Beth McDougall, Harriet Warburton, Lucy Shaw, and Kamal R. Mahtani in Journal of Applied Gerontology.
