# Supplementary material for: Understanding and Improving Older People’s Well-Being through Social Prescribing Involving the Cultural Sector: Interviews from a Realist Evaluation
Source: J Appl Gerontol. 2023 Feb 1;42(7):1466–76. doi: 10.1177/07334648231154043 (PMC10262332; doi:10.1177/07334648231154043)
Supplement: Supplemental Material—Understanding and Improving Older People’s Well-Being through Social Prescribing Involving the Cultural Sector: Interviews from a Realist Evaluation [file sj-pdf-1-jag-10.1177_07334648231154043.pdf]

Supplementary file 1: Key terms associated with realist research

Context: Influences whether a mechanism is triggered or not. It is the circumstances within which the intervention is delivered or executed and can relate to individuals (characteristics of stakeholders), interpersonal relationships, institutional factors (e.g. norms, culture) and infrastructure (wider sociocultural factors) (Pawson, 2013).

Mechanism: Underlying, often invisible causes of outcomes, "embodied in the subjects' reasoning…" (Pawson, 2013). Mechanisms are a response (e.g. fear, reputation management, feeling valued) to resources provided by an intervention.

Outcome: From a realist perspective, "it is not programs that 'work' but their ability to break into the existing chains of resources and reasoning [of individuals or groups]…" (Pawson & Tilley, 1997 p. 75). Hence, we were interested in variation in terms of how different outcomes were produced and patterns associated with this.

Context-mechanism-outcome configuration (CMOC): A proposition that the intervention produces an outcome (O) "because of the action of some underlying mechanisms (M), which only comes into operation in particular contexts (C)" (Pawson, 2013 p. 22). In their simplest form, CMOCs are statements or causal claims that explain how a specific context can activate certain mechanisms to produce a particular outcome.
