# Supplementary material for: Understanding and Improving Older People’s Well-Being through Social Prescribing Involving the Cultural Sector: Interviews from a Realist Evaluation
Source: J Appl Gerontol. 2023 Feb 1;42(7):1466–76. doi: 10.1177/07334648231154043 (PMC10262332; doi:10.1177/07334648231154043)
Supplement: Supplemental Material—Understanding and Improving Older People’s Well-Being through Social Prescribing Involving the Cultural Sector: Interviews from a Realist Evaluation [file sj-pdf-2-jag-10.1177_07334648231154043.pdf]

Supplementary file 2: Example questions asked in interviews (however, they were semi-structured so the questions asked varied depending on topics raised by participants)

Older people	***Cultural sector staff***
• How might places like museums, libraries and botanical gardens support people's health and well-being?<br>• What might stop older people using museums, libraries and botanical gardens?<br>• How can museums, libraries and botanical gardens be used to help people to connect to others?<br>• How might online cultural activities differ to those that take place in person?<br>• What do you understand by the term 'social prescribing'? How might museums, libraries and botanical gardens play a role in this?	• What is your organisation doing to support the well-being of older people?<br>• How has what you offer to the public changed/been affected by COVID-19?<br>• What connection, if at all, does your organisation have with social prescribing/LWs?<br>• What are the potential risks or negative consequences of organisations like your own attempting to support older people's well-being?<br>• How do you assess if what you are doing helps people's well-being?
