# Supplementary material for: Understanding and Improving Older People’s Well-Being through Social Prescribing Involving the Cultural Sector: Interviews from a Realist Evaluation
Source: J Appl Gerontol. 2023 Feb 1;42(7):1466–76. doi: 10.1177/07334648231154043 (PMC10262332; doi:10.1177/07334648231154043)
Supplement: Supplemental Material—Understanding and Improving Older People’s Well-Being through Social Prescribing Involving the Cultural Sector: Interviews from a Realist Evaluation [file sj-pdf-3-jag-10.1177_07334648231154043.pdf]

Supplementary file 3: CMOCs from our review and new (N) or revised (R) CMOCs from interview data

CMOCs from our realist review	*Revised (R)/new (N) CMOCs drawing on interview data*
**Messaging**	**Messaging = we refined this concept by incorporating ideas on perseverance and elitism**
• CMOC1: When a LW can provide detailed information about a cultural offer (C), the older person is more likely to understand if it is suitable for them (O) because they can work out what it entails (M).<br>• CMOC2: When the LW explains the cultural offer as part of social prescribing in a way that shows how it relates to an individual's needs (C) because it is regarded as a credible solution (M), the older person is more likely to accept it (O).	• CMOC1-N: When LWs warn older people in advance that it can take time to find an offer that they will get benefit(s) from (C), older people are more willing to continue trying new offers (O), because this is what they expect to happen (M).<br>• CMOC2-N: When a trusted person encourages an older person to keep attending an offer they did not like after their first attendance (C), they may re-attend (O), because they are persuaded to persevere (M).<br>• CMOC3-N: When LWs describe an offer in a way that makes it sound accessible (C), an older person is likely to give it a go (O) because they feel it is something that is for them (M).
**Matching**	**Matching = we refined this concept by incorporating ideas on buddying**
• CMOC3: When LWs understand the needs and expectations of an older person (C), they are more likely to	• CMOC4-N: When an older person is well-matched with a buddy they can relate to (C), they are more willing to

suggest a suitable cultural offer (O) because they have an understanding of what is acceptable to and needed by that individual (M).	attend an offer (O) because they have a bond and enjoy being with this individual (M).<br>• CMOC5-N: When a 'buddy' scheme is available (C), older people are more likely to attend a cultural offer (O) because it provides a sense of security (M).
**Monitoring**	**Monitoring = remained unchanged**
• CMOC5: If cultural institutions evaluate the cultural offers they make to older people (C), they can adapt the suitability of the offer (O) because they are aware of the changes needed (M).<br>• CMOC6: When a LW asks for feedback from older people attending cultural offers (C) they can assess whether a cultural offer is benefiting someone (M) and changes can be made to the individual's action plan if required (O).<br>• CMOC7: When LWs and cultural sector staff collaborate constructively (C), improvements to cultural offers are more likely (O) because their shared knowledge is harnessed (M).	
**Partnerships**	**Partnerships = remained unchanged**
• CMOC8: When a cultural organisation is committed to supporting public well-being (C), because staff feel that they are undertaking such work in a facilitative environment (M) they are	

willing to make changes and take risks (O).<br>• CMOC9: When older people are consulted about the content of cultural offers (C), something is developed by cultural organisations that is appropriate and acceptable to end users (O) because it has taken into consideration their ideas (M).<br>• CMOC10: When LWs and cultural sector staff interact (C), it allows for greater understanding and valuing of each party's contribution to older people's well-being (M), which promotes a willingness to collaborate (O).	
	**Maintaining boundaries = we developed this new concept to incorporate ideas on capacity and emotional involvement**
	• CMOC6-N: When cultural institutions have limited resources (C) staff may be uncertain about offering social prescribing activities (O) because they worry about not being able to offer people the right support (M).<br>• CMOC7-N: When a cultural offer has clear boundaries (C), cultural sector organisations may be more likely to develop offers (O), because they do not worry about user dependency and resource implications of open-ended offers (M).<br>• CMOC8-N: When cultural sector

	institutions provide staff and volunteers with emotional support structures (C), they are less likely to burnout (O) because they feel supported (M).
**Distracting**	**Immersing = we retitled this concept (from 'distracting') to reflect a less passive interaction**
• CMOC11: When an older person finds the cultural offer stimulating (C), they experience an escape from their problems (O) because they enjoy and are absorbed by the activity (M).<br>• CMCO12: When the cultural offer engages older people's senses (C), their enjoyment increases (O) because their mind is elsewhere (M).	• CMOC9-R: When older people attend a beautiful and well-tended space (C), they are afforded a sense of peace and relaxation (O) because their senses are being engaged (M).<br>• CMOC10-R: When older people attend a cultural venue with a quiet ambiance (C), they are distracted from worries (O) because they are immersed in their surroundings (M).
**Holding**	**Holding = we refined this concept by incorporating ideas on elitism**
• CMOC13: When the cultural environment is older people friendly (C) they enjoy attending (O) because they feel safe and at ease (M).<br>• CMOC14: When the cultural offer is delivered professionally and consistently (C), older people feel reassured (O) because they know what to expect (M).	• CMOC11-N: When an older person perceives that the venue or offer is elitist (C), they may be put off attending (O) because they do not feel welcomed or that it is for them (M).
**Connecting**	**Connecting = we refined this concept by incorporating ideas on café culture**

• CMOC15: When the cultural offer provides a social component (C), older people feel less lonely (O) because they have been facilitated to engage in human interactions (M).<br>• CMOC16: As the cultural offer continues to provide a social component (C), older people increase their social network (O) because they have been facilitated to develop and maintain new relationships (M).	• CMOC12-R: When a cultural offer provides older people with opportunities to socialise (C), they feel less lonely and more connected (O) because they have human interactions (M).<br>• CMOC13-N: When the venue has a nice café (C), older people feel connected (O) because they value the services and ambiance (M).
**Transforming**	**Transforming = we refined this concept by incorporating ideas on autonomy**
• CMOC17: When the cultural offer enables older people to experience or learn new things (C), their self-esteem and confidence increase (O) because they are encouraged to try things outside of their comfort zone (M).<br>• CMOC18: When older people are given the option to take part in a cultural offer in a way that suits their preferences (C), their self-worth is increased (O) because they feel attended to (M).	• CMOC14-N: When older people are able to engage with a cultural offer in ways of their choosing (C), they enjoy themselves (O) because they are able to exercise their autonomy (M).<br>• CMOC15-N: When older people are able to exercise personal choice (C), they may re-attend an offer even if they did not like it the first time (O) because they feel some degree of control (M).
	**Virtual cultural offers = we developed this as a new cross cutting concept (see Figure 1) based on data from interviews with older people**
	• CMOC16-N: When a virtual offer provides an older person with access to experiences they would not normally

	be able to have (C), they are likely to attend (O), because they value this opportunity (M).<br>• CMOC17-N: When the cultural sector is supported to provide virtual offers that are organised and delivered professionally (C), older people are more willing to accept them (O) because the experience is entertaining and enjoyable (M).<br>• CMOC18-N: When virtual offers are older people friendly (C), individuals are more willing to attend them (O) because they are perceived as accessible (M).
