# Supplementary material for: Understanding and Improving Older People’s Well-Being through Social Prescribing Involving the Cultural Sector: Interviews from a Realist Evaluation
Source: J Appl Gerontol. 2023 Feb 1;42(7):1466–76. doi: 10.1177/07334648231154043 (PMC10262332; doi:10.1177/07334648231154043)
Supplement: Supplemental Material—Understanding and Improving Older People’s Well-Being through Social Prescribing Involving the Cultural Sector: Interviews from a Realist Evaluation [file sj-pdf-4-jag-10.1177_07334648231154043.pdf]

Supplementary file 4: Supporting quotations for concepts in the data about tailoring of cultural offers
P = Older person interviewee; CS = Cultural sector interviewee

**Messaging**
"I think the problem with museums is we're not very good at telling people what we do. I think it's the whole thing that museums need to do is articulate why they are important and what the benefits are so that people can understand we do what we do" **CS09**<br><br>"But yeah, certainly some people are nervous about being in a space like this or feeling it's not for them, it's only for kind of intellectuals or it's only for qualified people. So, it's always been a part of ethic to really emphasise that that isn't the case; that this is for everybody." **CS14**<br><br>"One thing I thought was that I think that they (link workers) need to be aware of what the resources are themselves if they're not aware of those resources. I'm not sure how many clinical staff would be aware of… well that they could send people" **CS24**<br><br>"But that can be hard as well, failure if you do something and it's not right for you, not to think oh, it's either than I'm too old for this or I'm not fit enough for it or I'm somehow failing, I'm not intellectually cut out, whatever it may be, and it's very important I think not to feel a failure, that if you try things and they're not right for you, and I think people have to be encouraged not to feel that they're failing if not all activities, or just the things that don't give people pleasure." **P005**<br><br>"…if you just haven't been doing it all your life or you haven't had time to do it or your whole life's pattern has stopped you doing it, to actually trigger starting to do it late in life must be harder…I fear it is a class thing probably which is a bad thing to say but it feels like it, you meet the people that are like you most of the time in theatres and museums." **P008**<br><br>"I suppose they need to trust the recommendation – that it might actually benefit them…That it doesn't necessarily replace…any medication that they might need to be on. It's an addition to boost their overall wellbeing and health, and that there are great social benefits to doing these things which they might be reluctant to at first, but they will soon probably enjoy." **P016**<br><br>"Just an example from my own condition – with my MS, I suffer from something called neuropathic pain, and neuropathic pain is incredibly difficult to prescribe relief. I was prescribed endless different medications before… After about five years, we eventually hit on one that reduces the pain, and it's going to be the same for social prescribing, isn't it?...maybe you have to try things out a few times before you hit the right thing to bring on the sense of wellbeing..." **P023**

**Matching**
“I think it can be quite an intimidating place, it’s a very grand building. Oxford in particular has a lot of academic people I think and the average member of the public might feel quite intimidated by this quite austere frontage, but by the same token it is for everybody and whether you just come in and have a look around or you’re interested in something very specific, it’s just a nice place. You’ll always find something that catches your eye, you know, so I do think it could appeal to everybody but I can understand why, if you’re a little bit anxious, there’s a booking system in place now, if you haven’t understood that or are able to access that, that could be problematic.” **CS17**<br><br>“There's almost a bit of a caring role develops with some of them I think. Again that was really the case of COVID I know a number of library staff mentioned to me that they supported older customers who’d been bereaved during COVID and had these kinds of conversations with them as well.” **CS22**<br><br>“...it's very important for the majority of the volunteer team to have that connection. For the staff that are managing the volunteer team on a day-to-day basis, it can feel like an additional task. But equally you know it's the right thing to do...and you do the same for a fellow staff member as you would a fellow volunteer member. But it does mean that it can have an impact on your capacity for the rest of your role, absolutely.” **CS09**<br><br>“So I think it does need some sort of buddying up to give that person confidence to join a group… I mean other people would just go along but if people who are slightly lacking in confidence or if they’ve been socially prescribed, they feel a bit vulnerable I think because they feel they’re not as they should be you know in terms of their health or wellbeing and so I think it’s even more important in that context that there’s a buddying system.” **P010**<br><br>“I found things like reading groups that you discover in a reading group people who you get on well with and then suddenly one of them says I notice that you know, X author is appearing at X, do you wanna come with me and you know, you go together. So I think [a buddy scheme] would be useful… your link worker could of course pair up people who’ve been to – you know if it’s a museum, they could pair up somebody who’s reasonably confident about going to museum with somebody who’d never been to one before.” **P018**
**Monitoring**
“One thing I’d also like is – if social prescribers are out there meeting people, then they’ll know what people want – what the community needs… They’re dealing with them every day, so they know what the common conditions are – feelings are – so that would be really useful to get that kind of information more shared widely.” **CS09**

"The difficult thing is trying to get feedback and we have been experimenting with giving paper copies of feedback, but it's quite hard encouraging… people haven't been motivated to give that feedback. When we do the group in person, obviously, it's so much easier because you can see how things are going." **CS12**<br><br>"... I'd say absolutely for many people and we send out questionnaires evaluation forms after all our events and we ask the people what do you think of these events, would you like to see them." **CS23**
**Partnerships**
"I've been working with a doctor's surgery…and they're about five minutes' walk from [gallery]. They have actually got two social prescribers as members of staff. I've been working closely with the two social prescribers. It's very much a testing and exploring approach." **CS06**<br><br>"My instinct is it would probably, again because just the nature of the need of the people taking part, the cultural organisation is not the expert in that. Whereas the GP surgery is more likely to have the relevant skills and knowledge in order to make sure the participants have what they need…If someone taking part, for example if someone has mental health difficulties and there is a significant trigger for them, making sure the site is aware of that. If say there's building work and there's sudden loud noises…that could be a real trigger for them. That sharing of information without sharing too much personal information would be really important." **CS10**<br><br>"…when we have run activities with specific groups, for example, we've had adults with learning difficulties in before and having careers and a specific leader who is working in tandem with a member of the museum staff for example to deliver sessions, has been really beneficial, and that kind of project works well." **CS19**<br><br>"For us I think it can be off putting, people have actually said to us it's intimidating for many people from less affluent backgrounds that we're in the middle of a big art centre. So we tried to reach these people using different organisations that are already working with them so say social services, Age UK, Carers networks…" **CS22**<br><br>"Yeah pretty much everything we do we do in partnership yeah that's a key ingredient is having a clinical partner in helping us to set that up and then working with our artists to build their experience and skills." **CS25**

Maintaining boundaries
“My worry with that is the transition into offline, so always being clear about what the next step is. I think within a course, that’s easier to do. You can say at the end of this eight weeks, here are the options. You’ll be able to volunteer in the gardening project, join the walk and talk programme, so you’re not just saying to people, ‘off you go’…You can’t continue on a social prescribing project forever. You don’t want it to be a temporary fix that people feel better about for a short time and then it’s like now I’m back…” **CS06**<br><br>“The challenges are time and emotional energy, because with people who are really vulnerable and experiencing high anxiety or severe depression, you never quite know what the day will bring for them, you never quite know, once they trust you, what they’ll want to tell you…all the volunteer managers went on a bit of a journey with this because it was different from just managing volunteers who were fairly confident, self sufficient.” **CS08**<br><br>“But um I think the resource is probably the main thing, and having the staff members and the volunteers to support that kind of thing, would probably be the number one challenge.” **CS19**<br><br>“…we have finite scheduling for everybody, finite resource of people, so it’s not about cash. It’s where we deploy the people within our teams to work with audiences. And that kind of work is really valuable. It’s very labour intensive.” **CS15**<br><br>“So I can imagine, for example, programmes about working with people with dementia. I don’t know this for sure but I imagine that colleagues of mine at the [museum name] who were involved in those programmes had training or they brought in specialist facilitators and that all comes with costs and expertise, and I’m sure there’ll be plenty of museums whose staff wouldn’t feel skilled and that would require a training programme and a commitment, because you wouldn’t want to put all of that effort in to then only do a one-off event, you know, you’d feel like this would need to be something that we would want to develop over several years.” **CS20**
