# Supplementary material for: Understanding and Improving Older People’s Well-Being through Social Prescribing Involving the Cultural Sector: Interviews from a Realist Evaluation
Source: J Appl Gerontol. 2023 Feb 1;42(7):1466–76. doi: 10.1177/07334648231154043 (PMC10262332; doi:10.1177/07334648231154043)
Supplement: Supplemental Material—Understanding and Improving Older People’s Well-Being through Social Prescribing Involving the Cultural Sector: Interviews from a Realist Evaluation [file sj-pdf-5-jag-10.1177_07334648231154043.pdf]

Supplementary file 5: Supporting quotations for concepts in the data about the benefits of engaging with tailored cultural offers
P = Older person interviewee; CS = Cultural sector interviewee

**Immersing**
"You don't have to be looking at these works with any sense of – it can just be for pleasure, for enjoyment. That's a completely legitimate and important reason for looking at things. So, we don't make a distinction between visitors being more or less important depending on their type of interests. For us it's all one, so we really try to get that message across to people." **CS14**<br><br>"…walking around [gardens] it just refreshes one completely. I'm retired now, but before I retired, I worked within a university and I found that if I had a particularly stressful meeting or encounter or something like that, then getting out and walking around the campus would be a wonderful, refreshing thing to do. You always feel better after walking around in natural surroundings, so as far as gardens are concerned, it's that kind of enjoyment of nature combined with the restorative quality that nature can have, just rebalances you." **P007**<br><br>"I found it really nice, even though it was early in the season for gardens. Because of the booking entry which you could only go in so many people at a time, it was beautiful and quiet, and it was uplifting to see things coming through, which I thought in a month on it would look beautiful…I've always enjoyed the open spaces anyhow and being out and about and walking has always been very important to me… To walk into the city park from the city, it's like stepping out and it's just been lovely, really, really lovely. So, yes breathe in the fresh air." **P011**<br><br>"To me there's something about being around in a beautiful place. Last week I was in the [setting] with a friend and we were just looking at some of the paintings that we hadn't seen for a long time and we were wandering around, having a chat…When I'm in a really lovely garden like a National Trust garden, I just like the smell of it, the look of it, marvelling at the way somebody has planned it and designed it, worked on it. It's just a feeling of I don't know, it takes your brain away for a start from other things you might be thinking about." **P025**
**Holding**
"…we're based in [cultural organisation] so we have many [local area] residents who are very well off and educated but then we have a couple of the estates nearby where people are less well-off maybe less educated less confident and those people are less likely to sign up for an art class because they lack confidence I think and will feel it's not for them." **CS23**<br><br>"People need reassurance and reaffirmation, so I think it's worth persisting a bit. Maybe the old-fashioned leaflet for older people might be good, rather than it all being online so they have it in their hands that they can take home and read, and absorb, and feel reassured by – if you had the phone number of the buddy on it. That kind of thing can help, but I wouldn't give up at the first hurdle – I'd definitely keep

going.” **P016**<br><br>“The way the social prescribing works is the social prescriber identifies somebody and then I do a meet and greet session on the phone. Some people just don’t want to do Zoom at all and have been really put off and have said no. The people who have done it, actually really love it. If you’ve got any kind of social anxiety issues, weirdly they’re finding Zoom much easier because, as one person said, ‘you don’t have to go out, you can’t fall out with anybody’. These are her words. She never used Zoom before ever, but she said it feels like a safe space that doesn’t feel like the anxiety of going to a group.” **CS08**
**Connecting**
“I think elderly people…do seem to like that sense of community and group activities and I think they enjoy coming together and reminiscing, so I think rather than an individual coming to a museum, if it’s part of a group outing that’s probably the way to go, as long as they’ve all had that say of yes, that would be lovely, I’d really like to do that, and then match them all up together maybe…” **CS17**<br><br>“…[a café] is lovely, because again if you’re wandering around having a look, it’s nice just to have a sit down sometimes and then just carry on and make a morning of it or make a day of it.” **P015**<br><br>“I think it’s very good socially, actually, because my poor friends are very tolerant of my new discoveries, so when you meet your friends, you can say, ‘Hey, did you know that?’, and then usually they know something about it so I think it’s a very good connecting thing, actually, between… if you have like-minded friends which most of us do.” **P020**<br><br>“You just want to feel that you’re not isolated and lost...You do have feelings and needs for interaction with other people, and I think museums, gardens, and libraries can all help to promote that in a sort of quiet way.” **P028**
**Transforming**
“Just inclusive, collaborative practice, is that we just work in that way. So, you’re working together with people. So, it’s very much that – with people. And so, you’re designing, developing things with people, for people.” **CS15**<br><br>“With things like museums as well it’s a learning thing, and by doing that you’re sort of taking on the experiences. I think it gives you things to think about, which is always good I think, to have different things to think about and ideas to explore in your head and things like that, which is I think good for wellbeing because you’re not just looking in all the time about how you’re feeling and what your life’s like and things but

you’re looking outside” **P005**

“I often go to museums on my own. I like going to museums on my own, often, because I don’t have to… especially if you’re going to an exhibition, you can go around in your own time and you notice different things, I think, if you’re not with somebody.” **P006**

“I seriously think as you’re getting older, you should do this for your brain - all this myth that we’re told that your brain does atrophy, well I think we don’t quite believe it so we make a bit of an effort!” **P020**

“Walking about the space, or a venue – that’s important activity… it’s very stimulating – so there’s the activity. But this is the stimulation that you can’t get just by sitting and daydreaming or watching television – it’s nothing quite the same and particularly an insight that you get from professional curators with all this stuff. If you engage with it, you can’t really lose because, even if you say, ‘Oh, this is not worth it,’ which is rarely the case – you seem to get a lot out of it, and I think that stimulation is amazing. And, through being out there, you are more likely to meet other people that can bring different views, different perspectives, or even give you ideas about what you can do.” **P024**
