# Supplementary material for: Understanding and Improving Older People’s Well-Being through Social Prescribing Involving the Cultural Sector: Interviews from a Realist Evaluation
Source: J Appl Gerontol. 2023 Feb 1;42(7):1466–76. doi: 10.1177/07334648231154043 (PMC10262332; doi:10.1177/07334648231154043)
Supplement: Supplemental Material—Understanding and Improving Older People’s Well-Being through Social Prescribing Involving the Cultural Sector: Interviews from a Realist Evaluation [file sj-pdf-6-jag-10.1177_07334648231154043.pdf]

COREQ (COnsolidated criteria for REporting Qualitative research) Checklist

A checklist of items that should be included in reports of qualitative research

Topic	Item No.	Guide Questions/ Description	Author Responses
**Domain 1: Research team and reflexivity**			
Personal Characteristics			
Interviewer/facilitator	1	Which author/s conducted the interview or focus group?	This is described in the methods section
Credentials	2	What were the researcher's credentials? E.g. PhD, MD	Background of researchers is described in the methods section
Occupation	3	What was their occupation at the time of the study?	This information is in the methods, in the section on data collection
Gender	4	Was the researcher male or female?	This information is in the methods, in the section on data collection
Experience and training	5	What experience or training did the researcher have?	One has an MSc in anthropology and another has a PhD in medical anthropology
Relationship with participants			
Relationship established	6	Was a relationship established prior to study commencement?	Due to the collection of data during the pandemic, this was not possible
Participant knowledge of the interviewer	7	What did the participants know about the researcher? e.g. personal goals, reasons for doing the research	Participants were told that we were doing this study to try and understand how older people might benefit from using the cultural sector for health and well-being
Interviewer characteristics	8	What characteristics were reported about the inter-viewer/facilitator? e.g. Bias, assumptions, reasons and interests in the research topic	Some details have been reported in the methods section – the researchers conducting data collection and analysis were employed to work on the project
**Domain 2: Study design**			
Theoretical framework			
Methodological orientation and Theory	9	What methodological orientation was stated to underpin the study? e.g. grounded theory, discourse analysis, ethnography, phenomenology, content analysis	This was part of a realist evaluation
Sampling	10	How were participants selected? e.g. purposive, convenience,	Purposive sampling was undertaken

		consecutive, snowball	
Method of approach	11	How were participants approached? e.g. face-to-face, telephone, mail, email	They were contacted via email (though gatekeepers/contacts)
Sample size	12	How many participants were in the study?	25 cultural sector staff and 28 older people
Non-participation	13	How many people refused to participate or dropped out? Reasons?	No one dropped out or refused to get involved once they had contacted the researchers about taking part – the invitation may have been sent to a number of people who decided not to get involved and did not contact the research team – however, we do not have this information
Setting			
Setting of data collection	14	Where was the data collected? e.g. home, clinic, workplace	Data were collected via telephone or Microsoft Teams
Presence of non-participants	15	Was anyone else present besides the participants and researchers?	No
Description of sample	16	What are the important characteristics of the sample? e.g. demographic data, date	Older people (aged 60 or older) and cultural sector providers
Data collection			
Interview guide	17	Were questions, prompts, guides provided by the authors? Was it pilot tested?	We provide some details of questions asked<br>Questions were shared with our patient-public involvement group in advance of conducting interviews
Repeat interviews	18	Were repeat interviews carried out? If yes, how many?	No
Audio/visual recording	19	Did the research use audio or visual recording to collect the data?	Interviews were audio-recorded with participants' consent
Field notes	20	Were field notes made during and/or after the interview or focus group?	No
Duration	21	What was the duration of the interviews or focus group?	Interviews lasted between 30-60 minutes
Data saturation	22	Was data saturation discussed?	The sample size was informed, to some extent, by the time we had available to conduct this project - we feel that towards the end of data collection we were not learning

			vastly new things to inform the programme theory, so had reached a point of data redundancy
Transcripts returned	23	Were transcripts returned to participants for comment and/or corrections?	No
**Domain 3: analysis and findings**			
Data analysis			
Number of data coders	24	How many data coders coded the data?	Two researchers were involved in the intensive coding stage - they shared their ideas with the project leads (3 other people) and then the wider research team (all authors) for comment and feedback
Description of the coding tree	25	Did authors provide a description of the coding tree?	We describe how context-mechanism-outcome configurations were developed as this was part of a realist evaluation
Derivation of themes	26	Were themes identified in advance or derived from the data?	There was deductive and inductive coding involved as we had a programme theory from a previous realist review, but inductive coding was used when parts of the data did not fit elements from this previous programme theory
Software	27	What software, if applicable, was used to manage the data?	NVIVO
Participant checking	28	Did participants provide feedback on the findings?	No
Reporting			
Quotations presented	29	Were participant quotations presented to illustrate the themes/findings? Was each quotation identified? e.g. participant number	Yes
Data and findings consistent	30	Was there consistency between the data presented and the findings?	Yes
Clarity of major themes	31	Were major themes clearly presented in the findings?	Yes
Clarity of minor themes	32	Is there a description of diverse cases or discussion of minor themes?	We present the main concepts that were incorporated into a revised programme theory as this work was

			related to a realist evaluation

Developed from: Tong A, Sainsbury P, Craig J. Consolidated criteria for reporting qualitative research (COREQ): a 32-item checklist for interviews and focus groups. *International Journal for Quality in Health Care*. 2007. Volume 19, Number 6: pp. 349 – 357
